# Higher educational attainment associated with reduced likelihood of abnormal cervical lesions among Zambian women - a cross sectional study

**DOI:** 10.1186/s12885-017-3680-z

**Published:** 2017-10-13

**Authors:** Twaambo Euphemia Hamoonga, Rosemary Ndonyo Likwa, Patrick Musonda, Charles Michelo

**Affiliations:** 1 Department of Global Health, Population Studies Unit, School of Public Health, University of Zambia, PO Box 50110, Lusaka, Zambia; 2 Department of Epidemiology & Biostatistical Unit, School of Public Health, University of Zambia, Lusaka, Zambia; 3 University of Bergen, Center for International Health, Bergen, Norway

**Keywords:** Zambia, Abnormal cervical lesions, cervical cancer, Education, Women

## Abstract

**Background:**

The high burden of cervical cancer in Zambia prompted the Ministry of Health and partners to develop the cervical cancer prevention program in Zambia (CCPPZ) in 2006. Despite this intervention more women continue to die from the disease and there is little understanding of factors that may be linked with abnormal cervical lesions in the general population. We therefore examined if educational attainment is associated with abnormal cervical lesions among Zambian women aged 15 to 49 years.

**Methods:**

This study used data from the cervical cancer prevention program in Zambia, where a total of 14,294 women aged 15 to 49 years were screened for cervical cancer at nine health facilities between October 2013 and September 2014. The data represents women from six provinces of Zambia, namely Southern, Central, Copperbelt, Luapula, North-western and Eastern provinces. Step-wise logistic regression analysis using the Statistical Package for the Social Sciences (SPSS) version 21 was used to estimate adjusted odds ratios (AOR) and 95% confidence intervals (CIs) for educational attainment with presence of abnormal cervical lesions as outcome. Multiple imputation was further used to obtain the imputed stabilized estimates for educational attainment.

**Results:**

The prevalence of abnormal cervical lesions, using the Visual Inspection with Acetic-acid (VIA) test was 10.7% (*n* = 1523). Educational attainment was inversely associated with abnormal cervical lesions (AOR = 0.75; 95% CI:0.70–0.81, AOR = 0.74; 95% CI:0.68–0.81 and AOR = 0.46; 95% CI:0.41–0.51) among women with primary, secondary and tertiary education, respectively, compared to those with no formal education.

**Conclusion:**

We find reduced likelihood of abnormal cervical lesions in educated women, suggesting a differential imbalance with women who have no formal education. These findings may be a reflection of inequalities associated with access to cervical cancer screening, making the service inadequately accessible for lower educated groups. This might also indicate serious limitations in awareness efforts instituted in the formative phases of the program. These findings underline the prevailing need for urgent concerted efforts in repackaging cervical cancer awareness programs targeting women with low or no formal education in whom the risk may be even higher.

## Background

Reproductive health needs increase during adolescence and reproductive years, particularly for women, and in later years the general health continues to reflect earlier reproductive life events with other health issues such as cancers becoming more prominent [1]. Non-communicable diseases and cancers, are recognized as an increasing problem globally, especially for low and middle income countries [2].

Cervical cancer (CC) is the second most common female malignancy in the world [3]. Worldwide, approximately 493,000 new cases of CC are diagnosed annually [4]. About 80 to 85 % of these cases are in developing countries [5], reflecting limited access to health care and preventive technologies. Cervical cancer is the second most frequently diagnosed cancer (80,400 cases) and the leading cause of cancer deaths (50,300), which is approximately 62.6% of all those who are diagnosed with the disease in Africa [6]. The 2013 consensus paper on the recommendations for the prevention of cervical cancer in sub-Saharan Africa asserts that more than 200 million females older than 15 years are at risk in this region. Some countries in East and Southern Africa, including Zambia, Malawi, Mozambique, and Tanzania have among the highest worldwide cervical cancer rates (50 cases per 100,000) [2]. The standardized cervical cancer incidence rate for Zambia is above 55 per 100,000 whereas the standardized mortality from cancer of the cervix stands at 41 per 100,000, making Zambia’s cancer burden only second in Africa after Guinea and 6th in the world [7].

For many years now, cervical cancer has continued to claim the lives of many women in Zambia with 80% of cases being advanced at presentation, when only palliative treatment can be given [8]. This prompted the Ministry of Health and partners to launch the “Cervical Cancer Prevention Program in Zambia” (CCPPZ) in 2006. The CCPPZ, which in its initial phase of implementation was only targeting the highest risk HIV-infected women, has cumulatively provided services to over 58,000 women (regardless of HIV status) over the past 5 years [7]. However, studies have shown that advances in cancer treatment have not been as effective as those for other chronic diseases with respect to reducing mortality [9]. Therefore, a shift towards addressing risky sexual behavior, especially that which exposes women to HPV, would prevent a substantial proportion of deaths from the disease. It is assumed that diseases like cervical cancer are only properly estimated and managed when populations understand the factors that may be associated with them. We argue that this understanding can only be effective if literacy levels are high. One proxy associated with literacy is educational attainment and in as far as cervical cancer programs are concerned understanding of this link is limited. We thus determined the prevalence of abnormal cervical lesions and the possible association with educational attainment among Zambian women aged 15–49 years old.

## Methods

### Cervical cancer prevention program in Zambia

The Cervical Cancer Prevention Program in Zambia (CCPPZ), is a program that was launched in 2006 and has continued to provide screening services to women at 26 government health facilities in 14 districts in nine (9) provinces, namely Southern, Central, Northwestern, Luapula, Northern, Copperbelt, Eastern, Western and Lusaka provinces.

The CCPPZ was designed to increase access to cervical cancer screening in order to reduce the incidence and prevalence of the disease through screening using visual inspection with dilute (5%) acetic acid (VIA) linked to immediate cryotherapy (see and treat).

### Cervical cancer and education design

This was a cross-sectional study which utilized secondary data that was collected under the CCPPZ. The population for the study comprised 14,294 women aged 15–49 years old who had ever been screened for cervical cancer at one of nine (9) selected government health facilities whose data for the study period (October 2013 and September 2014) was up to date. The dataset that was used for this study was collected from the Centre for Infectious Disease Research in Zambia (CIDRZ), which hosts the main database for all the centers offering cervical cancer screening under the CCPPZ.

### Data extraction

Using the CCPPZ database, only data for those women whose records had the outcome of the screening stated as either VIA positive or VIA negative for presence of abnormal cervical lesions was extracted to define the sampling frame. In this study, a VIA positive result represented an abnormal cervical lesion, where an abnormal cervical lesion was defined as an acetowhite lesion or whitish patch on the uterine cervix when ‘painted’ or ‘stained’ with 5% acetic acid-vinegar. This variable together with complete information on the educational attainment status defined the de facto eligible sample for this study. Among the records of the de facto eligible sample, the information recorded and extracted included the women’s demographic characteristics such as age at screening, marital status, screening center/health facility, household income, occupation and highest level of educational attainment. Educational attainment was categorized into four categories: no formal education (those who had never been to school); primary education (both those who had acquired some level of primary education and those that had completed primary education- Grades 1–7); secondary education (both those that had acquired some level of secondary education and those that had completed secondary education- Grades 8–12); and tertiary education (both those that had acquired some level of tertiary education and those that had completed tertiary education from either a college or university). According to the 2014 National Education Profile for Zambia, on average, primary school attenders comprise of individuals whose ages range from 7 to 13 years and secondary school-goers ranging from 13 to 18 years old, after which one would be ready for tertiary education (UNESCO Institute for Statistics, 2014).

### Data analysis

Our study used both complete case analysis and multiple imputation, where the latter was used to assess whether the missing data, if imputed, could affect the association of educational attainment and abnormal cervical lesions observed from the complete case multiple logistic regression analysis. Using a chained multiple imputation approach as given by the mi stata command which uses Bayesian estimating procedure, a monotone uniform prior to do the multiple imputation was used with a burn in of 100 iterations and then 1000 iterations for the estimation.

All continuous variables were converted to categorical variables based on literature that was reviewed. This was done in order to allow for comparison of study findings to those from similar studies.

For data analysis, both descriptive and analytical statistical methods were used. The main predictor was educational attainment and the outcome was abnormal cervical lesion. The adjustment variables were chosen based on *p*-values from univariate logistic regression with abnormal cervical lesion as outcome using a significance level of 10%. The key estimates were unadjusted odds ratios (UOR) and adjusted odds ratios (AOR) for education. Adjusted odds ratios and 95% confidence intervals were estimated to evaluate educational attainment with presence of abnormal cervical lesions while adjusting for potential confounders. The Statistical Package for the Social Science (SPSS) version 21 was used for analysis of data and the significance level was set to 5%.

### Ethical considerations

Our study was approved by the Research Ethics and Science (ERES) Converge committee (Reference number: 2014-May-028) in Zambia. No written consent was obtained from participants as the study used secondary data and hence had no direct contact with them. However, permission to use the CCPPZ dataset was sought from the Director-CIDRZ, and approval to conduct the research was obtained from the University of Zambia (UNZA) School of Medicine.

## Results

Data used in the study represented women who were screened for cervical cancer at Choma General Hospital, Kasama General Hospital, Kitwe Central Hospital, Livingstone General Hospital, Mansa General Hospital, Mosi-oa-tunya Clinic, Ndola Central Hospital, Solwezi General Hospital, and St. Francis’ Hospital. This data represents a total of 9 screening facilities and these 9 screening facilities represent 6 out of the 10 provinces of Zambia. The study population comprised of women aged 15 to 49 years old (18.5% aged 15–24, 38.2% aged 25–34 and 43.3% aged 35–49). From a total of 14,294 participants whose VIA test results were known, 12,771 (89.3%) tested negative while 1523 (10.7%) women tested positive for abnormal cervical lesions. Table 1 depicts the descriptive statistics for the study population, stratified by whether participants tested positive or negative for abnormal cervical lesions.

**Table 1:** Socio-demographic characteristics of the study population

Variable	VIA Positive n (%)	VIA Negative n (%)	*P*-value
Age at screening (Valid 11,554, Missing 2740)			
15–24	205 (9.6)	1926 (90.4)	0.09
25–34	466 (10.5)	3954 (89.5)	
35+	568 (11.4)	4435 (88.6)	
Marital status (Valid 13,858, Missing 436)			
Never been married	186 (10.6)	1564 (89.4)	< 0.001
Married	942 (9.7)	8802 (90.3)	
separated, widowed, divorced	358 (15.1)	2006 (84.9)	
Educational attainment (Valid 13,843, Missing 451)			
No formal education	146 (12.9)	985 (87.1)	< 0.001
Primary education	574 (11.3)	4509 (88.7)	
Secondary education	548 (10.8)	4528 (89.2)	
Tertiary education	216 (8.5)	2337 (91.5)	
Household income (Valid 8666, Missing 5628)			
Less than K100	63 (8.7)	658 (91.3)	< 0.001
K100-K499	44 (17.1)	213 (82.9)	
K500-K999	112 (18.4)	498 (81.6)	
K1000-K5000	179 (13.8)	1117 (86.2)	
Above K5000	621 (10.7)	5161 (89.3)	
Occupation (Valid 12,848, Missing 1446)			
House wife	641 (11.4)	4963 (88.6)	<0.001
Formal employment	183 (8.7)	1913 (91.3)	
Informal employment	325 (9.3)	3172 (90.7)	
Other	233 (14.1)	1418 (85.9)	
Screening center (Valid 14,294, Missing 0)			
Choma General Hospital	40 (6.2)	608 (93.8)	< 0.001
Kasama General Hospital	74 (4.0)	1784 (96.0)	
Kitwe Central Hospital	243 (18.6)	1065 (81.4)	
Livingstone General Hospital	135 (5.9)	2136 (94.1)	
Mansa General Hospital	312 (24.7)	951 (75.3)	
Mosi-oa-tunya Clinic	61 (10.1)	543 (89.9)	
Ndola Central Hospital	303 (15.5)	1658 (84.5)	
Solwezi General Hospital	178 (6.9)	2398 (93.1)	
St. Francis’ Hospital	177 (9.8)	1628 (90.2)	

As shown in Table 1, statistics obtained from cross tabulations between the VIA test and the various socio-demographic characteristics of the study population revealed that in all age categories, there were more women testing negative for abnormal cervical lesions than those who tested positive. However, there was a higher percentage of women who tested positive for abnormal cervical lesions among the oldest age group of 35–49 years at 11.4%, compared to 9.6% among those aged 15–24 years old. Fewer women with tertiary education tested positive for abnormal cervical lesions (8.5%) compared to the other categories.

Women falling in the lowest and highest income categories were less likely to test positive for abnormal cervical lesions compared to women in the middle income category. With regards to marital status, results show a relatively higher proportion of married and never married women who tested negative for abnormal cervical lesions than those who were either separated, widowed or divorced.

Cross tabulations from Table 2 show that fewer women who reported having ever smoked cigarette tested positive for abnormal cervical lesions. There were more women who had their first sexual intercourse and first pregnancy below the age of 20 compared to their counterparts aged 20 years and above. Women who never used a condom with their regular sexual partners had the highest proportion of abnormal samples.

**Table 2:** Risk factors likely associated with abnormal cervical lesions

Variable	VIA Positive n (%)	VIA Negative n (%)	*P*-value
Ever smoke cigarette (Valid 13,350, Missing 944)			
yes	62 (18.1)	281 (81.9)	< 0.001
No	1372 (10.5)	11,635 (89.5)	
Ever used oral contraceptives (Valid 3536, Missing 10,758)			
Yes	389 (11.1)	3104 (88.9)	0.39
No	3 (7.0)	40 (93.0)	
Years on oral contraceptives (Valid 3035, Missing 11,259)			
One	104 (11.5)	799 (88.5)	0.99
Tow	187 (11.6)	1428 (88.4)	
Three	61 (11.8)	456 (88.2)	
Family history of cervical cancer (Valid 13,362, Missing 932)			
Yes	39 (11.7)	293 (88.3)	0.58
No	1405 (10.8)	11,625 (89.2)	
Number of pregnancies (Valid 12,957, Missing 1337)			
Never been pregnant	21 (8.3)	232 (91.7)	0.18
1–3 pregnancies	618 (11.4)	4787 (88.6)	
4 or more pregnancies	784 (10.7)	6515 (89.3)	
Age at 1st pregnancy (Valid 12,160, Missing 2134)			
Less than 20 years	837 (11.6)	6351 (88.4)	0.08
20+ years	528 (10.6)	4444 (89.4)	
Age at sexual debut (Valid 13,398, Missing 896)			
Less than 20 years	1141 (11.2)	9088 (88.8)	0.03
20+ years	311 (9.8)	2858 (90.2)	
Number of sexual life partners (Valid 13,660, Missing 634)			
One	392 (9.1)	3929 (90.9)	<0.001
Two	420 (10.9)	3446 (89.1)	
Three or four	467 (11.5)	3580 (88.5)	
5 or more	194 (13.6)	1232 (86.4)	
Condom use with regular sexual partner (Valid 11,774, Missing 2520)			
Never used a condom	775 (11.1)	6237 (88.9)	0.06
Used a condom sometimes	467 (11.4)	3627 (88.6)	
Used a condom almost all the time	48 (15.9)	253 (84.1)	
Always used a condom	38 (10.4)	329 (89.6)	
HIV status (Valid (11,118, Missing 3176)			
Positive	411 (16.6)	2063 (83.4)	< 0.001
Negative	759 (8.8)	7885 (91.2)	

Cross tabulations were further used to assess possible associations between our key exposure variable (education) and other predictor variables. Table 3 shows that among women aged 35 years and above, the majority of them had only acquired primary education while women younger than 35 years (both 15–24 and 25–34) had attained secondary education. Among the married, widowed, separated and divorced women, the highest level of educational attainment was primary while secondary education was the highest level attained among women who had never been married. Women with tertiary education constituted the highest proportion of those that had their sexual debut late (above 20 years old). Among women in formal employment, the highest proportion (71.8%) had acquired tertiary education while among house wives the majority (47.0%) only had primary education. The majority of women who reported having always used a condom with their regular sexual partners were those that had attained secondary education followed by those with tertiary education. The same observation was made among women who reported having used a condom almost all the time with their regular sexual partners.

**Table 3:** Cross tabulations: Educational attainment and other predictor variables

	Educational Attainment				
Variable	None	Primary	Secondary	Tertiary	*P*-value
Age at screening (Valid 11,229, Missing 3065)					
15–24	56 (2.7)	470 (22.7)	1158 (56.1)	382 (18.5)	< 0.001
25–34	221 (5.1)	1182 (27.5)	1779 (41.4)	1118 (26.0)	
35+	386 (7.9)	2187 (45.0)	1529 (31.4)	761 (15.6)	
Marital status (Valid 13,624, Missing 670)					
Never been married	35 (2.0)	145 (8.4)	858 (49.7)	687 (39.8)	< 0.001
Married	781 (8.2)	3904 (40.8)	3352 (35.0)	1538 (16.1)	
separated, widowed, divorced	290 (12.5)	980 (42.2)	782 (33.6)	272 (11.7)	
Household income (Valid 8543, Missing 5751)					
Less than K100	96 (13.8)	320 (45.9)	240 (34.4)	41 (5.9)	< 0.001
K100-K499	48 (19.0)	138 (54.5)	52 (20.6)	15 (5.9)	
K500-K999	45 (7.5)	322 (53.8)	183 (30.6)	48 (8.0)	
K1000-K5000	62 (4.9)	617 (48.6)	456 (35.9)	134 (10.6)	
Above K5000	148 (2.6)	1580 (27.6)	2289 (40.0)	1709 (29.8)	
Occupation (Valid 12,461, Missing 1653)					
House wife	648 (11.8)	2579 (47.0)	2046 (37.3)	214 (3.9)	<0.001
Formal sector	14 (0.7)	141 (6.8)	426 (20.7)	1481 (71.8)	
Informal sector	148 (4.3)	1328 (38.4)	1585 (45.8)	396 (11.5)	
Other	145 (8.9)	482 (29.5)	640 (39.1)	368 (22.5)	
Screening center (Valid 13,843, Missing 451)					
Choma General Hospital	35 (5.4)	225 (34.8)	303 (46.8)	84 (13.0)	< 0.001
Kasama General Hospital	92 (5.0)	792 (42.9)	798 (43.3)	163 (8.8)	
Kitwe Central Hospital	39 (3.0)	320 (24.5)	494 (37.9)	451 (34.6)	
Livingstone General Hospital	186 (8.5)	784 (35.8)	856 (39.0)	367 (16.7)	
Mansa General Hospital	69 (5.6)	554 (45.1)	376 (30.6)	230 (18.7)	
Mosi-oa-tunya Clinic	10 (1.7)	149 (24.8)	279 (46.5)	162 (27.0)	
Ndola Central Hospital	65 (3.4)	557 (28.9)	704 (36.5)	603 (31.3)	
Solwezi General Hospital	185 (8.1)	895 (39.0)	805 (35.1)	411 (17.9)	
St. Francis’ Hospital	450 (25.0)	807 (44.8)	461 (25.6)	82 (4.6)	
Ever smoked cigarette (Valid 13,029, Missing 1265)					
yes	37 (10.9)	140 (41.1)	113 (33.1)	51 (15.0)	0.04
No	1038 (8.2)	4610 (36.3)	4674 (36.8)	2366 (18.6)	
Ever used oral contraceptives (Valid 3497, Missing 10,797)					
Yes	177 (5.1)	1153 (33.4)	1360 (39.4)	765 (22.1)	0.06
No	2 (4.8)	10 (23.8)	25 (59.5)	5 (11.9)	
Years on oral contraceptives (Valid 3014, Missing 11,280)					
One	51 (5.7)	290 (32.3)	34 (38.1)	214 (23.9)	0.05
Two	75 (4.7)	565 (35.2)	628 (39.2)	335 (20.9)	
Three	30 (5.8)	147 (28.6)	203 (39.5)	134 (26.1)	
Family history of cervical cancer (Valid 13,057, Missing 1237)					
Yes	15 (4.5)	79 (23.8)	118 (35.5)	120 (36.1)	< 0.001
No	1029 (8.1)	4707 (37.0)	4701 (36.9)	2291 (18.0)	
Number of pregnancies (Valid 12,610, Missing 1684)					
Never been pregnant	3 (1.2)	39 (16.2)	111 (46.1)	88 (36.5)	< 0.001
1–3 pregnancies	188 (3.6)	1248 (23.7)	2401 (45.6)	1423 (27.1)	
4 or more pregnancies	889 (12.5)	3592 (50.5)	2060 (29.0)	568 (8.0)	
Age at 1st pregnancy (Valid 11,869, Missing 2425)					
Less than 20 years	777 (11.1)	3382 (48.3)	2404 (34.3)	436 (6.2)	< 0.001
20+ years	161 (3.3)	1180 (24.2)	1980 (40.7)	1549 (31.8)	
Age at sexual debut (Valid 13,089, Missing 1205)					
Less than 20 years	885 (8.9)	4135 (41.5)	3740 (37.5)	1214 (12.2)	< 0.001
20+ years	100 (3.2)	606 (19.5)	1167 (37.5)	1242 (39.9)	
Number of sexual life partners (Valid 13,345, Missing 949)					
One	437 (10.3)	1661 (39.2)	1379 (32.5)	762 (18.0)	< 0.001
Two	292 (7.8)	1341 (35.7)	1404 (37.4)	720 (19.2)	
Three or four	244 (6.2)	1350 (34.2)	1575 (39.9)	777 (19.7)	
5 or more	71 (5.1)	598 (42.6)	598 (42.6)	228 (16.3)	
Condom use with regular sexual partner (Valid 11,533, Missing 2761)					
Never used a condom	835 (12.2)	3073 (45.0)	2100 (30.8)	816 (12.0)	< 0.001
Used a condom sometimes	119 (2.9)	1049 (25.9)	1780 (44.0)	1097 (27.1)	
Used a condom almost all the time	5 (1.7)	68 (22.7)	134 (44.8)	92 (30.8)	
Always used a condom	6 (1.6)	68 (18.6)	166 (45.5)	125 (34.2)	
HIV status (Valid (10,780, Missing 3514)					
Positive	104 (4.3)	815 (33.7)	1071 (44.3)	426 (17.6)	< 0.001
Negative	737 (8.8)	3036 (36.3)	2948 (35.2)	1643 (19.6)	

Using univariate logistic regression, educational attainment was negatively associated with abnormal cervical lesions (Table 4). Women who had attained secondary and tertiary education had a reduced risk of abnormal cervical lesions (UOR = 0.82; 95% CI:0.67–0.99 and UOR = 0.62; 95% CI:0.50–0.78), respectively, compared to women who had no formal education.

**Table 4:** Logistic Regression: Unadjusted and Adjusted Odds Ratios

Variable	UOR (95% CI)	*P*-value	** AOR (95% CI)	*P*-value
Age at screening - Complete cases: 11,554 (80.8%)				
15–24	1.00		1.00	
25–34	1.11 (0.93–1.32)	0.25	0.85 (0.65–1.12)	0.25
35+	1.20 (1.02–1.42)	0.03	0.83 (0.63–1.09)	0.18
Marital status - Complete cases: 13,858 (96.9%)				
Never been married	1.00		1.00	
Married	0.90 (0.76–1.06)	0.21	0.71 (0.51–0.98)	0.04
Separated, widowed, divorced	1.50 (1.24–1.81)	<0.001	0.85 (0.59–1.23)	0.39
Educational attainment – Complete cases: 13,843 (96.8%)				
No formal Education	1.00		1.00	
Primary Education	0.86 (0.71–1.04)	0.13	0.74 (0.52–1.07)	0.11
Secondary Education	0.82 (0.67–0.99)	0.04	0.74 (0.51–1.07)	0.11
Tertiary	0.62 (0.50–0.78)	<0.001	0.48 (0.30–0.77)	0.002
Household income – Complete cases: 8666 (60.6%)				
Less than K100	1.00			
K100-K499	2.16 (1.43–3.27)	<0.001	*	*
K500-K999	2.35 (1.69–3.27)	<0.001		
K1,000-K5,000	1.67 (1.24–2.27)	<0.001		
Above K5,000	1.26 (0.96–1.65)	0.10		
Occupation – Complete cases: 12,848 (89.9%)				
House wife	1.00		1.00	
Formal employment	0.74 (0.62–0.88)	0.001	0.92 (0.66–1.28)	0.61
Informal employment	0.79 (0.69–0.91)	0.001	0.73 (0.55–0.95)	0.02
Other	1.27 (1.08–1.50)	0.003	1.01 (0.75–1.35)	0.97
Screening center – Complete cases: 14,294 (100%)				
Choma General Hospital	1.00		1.00	
Kasama General Hospital	0.63 (0.43–0.94)	0.02	0.64 (0.35–1.17)	0.15
Kitwe Central Hospital	3.47 (2.45–4.92)	<0.001	4.16 (2.46–7.03)	<0.001
Livingstone General Hospital	0.96 (0.67–1.38)	0.83	1.46 (0.75–2.83)	0.26
Mansa General Hospital	4.99 (3.53–7.04)	<0.001	6.86 (4.11–11.44)	<0.001
Mosi-oa-tuntya Clinic	1.71 (1.13–2.59)	0.01	1.51 (0.80–2.84)	0.21
Ndola Central Hospital	2.78 (1.97–3.91)	<0.001	3.91 (2.38–6.42)	<0.001
Solwezi General Hospital	1.13 (0.79–1.61)	0.50	1.65 (0.95–2.88)	0.08
St. Francis’ Hospital	1.65 (1.16–2.36)	0.01	1.68 (1.00–2.83)	0.05
Ever smoked cigarette – Complete cases: 13,350 (93.4%)				
No	1.00		1.00	
Yes	1.87 (1.41–2.48)	<0.001	1.34 (0.86–2.07)	0.19
Age at 1st pregnancy – Complete cases: 12,160 (85.1%)				
Less than 20 years (Adolescents)	1.00		1.00	
20+ years	0.90 (0.80–1.01)	0.08	0.83 (0.67–1.04)	0.10
Age at sexual debut – Complete cases: 13,398 (93.7%)				
Less than 20 years	1.00		1.00	
20+ years	0.87 (0.76–0.99)	0.03	1.15 (0.89–1.49)	0.27
Number of sexual life partners - Complete cases: 13,660 (95.6%)				
5 or more	1.00		1.00	
One sexual partner	0.63 (0.53–0.76)	<0.001	0.83 (0.62–1.12)	0.22
Two sexual partners	0.77 (0.65–0.93)	0.01	0.98 (0.74–1.29)	0.87
three or four	0.83 (0.69–0.99)	0.04	0.97 (0.75–1.26)	0.84
Condom use with regular sexual partner – Complete cases: 11,774 (82.4%)				
Always used a condom	1.00		1.00	
Never used a condom	1.08 (0.76–1.52)	0.68	1.15 (0.70–1.90)	0.57
Used a condom sometimes	1.12 (0.79–1.58)	0.54	1.38 (0.84–2.26)	0.20
Used a condom almost all the time	1.64 (1.04–2.36)	0.03	1.64 (0.89–3.02)	0.12
HIV status – Complete cases: 11,118 (77.8%)				
Negative	1.00		1.00	
Positive	2.07 (1.82–2.36)	<0.001	1.91 (1.57–2.33)	<0.001

5917 (41.4%) complete cases were included in multiple logistic regression analysis

*The variable was not included in multivariate logistic regression analysis as it had a lot of missing data (39.4%)

**Adjusted odds ratios: Adjustment variables; age at screening, marital status, education, screening center, occupation, cigarette smoking, age at sexual debut, number of sexual life partners, condom use with regular sexual partner and HIV status

Multivariate regression was used to further evaluate educational attainment while adjusting for potential confounders. In multivariate analysis (Table 4), educational attainment still continued to be inversely associated with abnormal cervical lesions (tertiary vs. no formal education) (AOR = 0.48; 95% CI:0.30–0.77). Women who had attained tertiary education had a reduced risk of having abnormal cervical lesions compared to those with no formal education.

Results obtained from multiple imputation analysis show that educational attainment continued to be inversely associated with abnormal cervical lesions. Women who had attained primary, secondary and tertiary education had reduced odds of abnormal cervical lesions (AOR = 0.75; 95% CI:0.70–0.81, AOR = 0.74; 95% CI:0.68–0.81 and AOR = 0.46; 95% CI:0.41–0.51), respectively. While results from the complete case analysis showed that acquiring tertiary education was the only level of educational attainment that was statistically protective, the multiple imputed stabilized estimates (Table 5) show that attaining primary and secondary education equally significantly reduced the likelihood of abnormal cervical lesions among Zambian women. Suffice to mention that results from the two analyses do not contradict each other, as both show a statistically significant association between education and abnormal cervical lesions, except that the latter provides improved statistical precision as can be noted from the confidence intervals that are narrower than those obtained from the complete case analysis. The multiple imputed stabilized estimates are shown in Table 5.

**Table 5:** Complete Case and Multiple Imputed Estimates

Variable	^b^ AOR (95% CI)	*P*-value	^c^ AOR (95% CI)	*P*-value
Age at screening (^a^ 2740)				
15–24	1.00		1.00	
25–34	0.85 (0.65–1.12)	0.25	1.02 (0.95–1.08)	0.64
35+	0.83 (0.63–1.09)	0.18	0.94 (0.74–1.19)	0.05
Marital status (^a^ 436)				
Never been married	1.00		1.00	
Married	0.71 (0.51–0.98)	0.04	0.78 (0.73–0.84)	<0.001
Separated, widowed, divorced	0.85 (0.59–1.23)	0.39	0.98 (0.90–1.06)	0.54
Educational attainment (^a^ 451)				
No formal Education	1.00		1.00	
Primary Education	0.74 (0.52–1.07)	0.11	0.75 (0.67–0.81)	<0.001
Secondary Education	0.74 (0.51–1.07)	0.11	0.74 (0.68–0.81)	<0.001
Tertiary	0.48 (0.30–0.77)	0.002	0.46 (0.41–0.51)	<0.001
Occupation (^a^ 1446)				
House wife	1.00		1.00	
Formal employment	0.92 (0.66–1.28)	0.61	0.90 (0.84–0.98)	0.01
Informal employment	0.73 (0.55–0.95)	0.02	0.85 (0.80–0.90)	<0.001
Other	1.01 (0.75–1.35)	0.97	0.97 (0.91–1.04)	0.44
Screening center (^a^ n/a)				
Choma General Hospital	1.00		1.00	
Kasama General Hospital	0.64 (0.35–1.17)	0.15	0.75 (0.65–0.88)	<0.001
Kitwe Central Hospital	4.16 (2.46–7.03)	<0.001	4.88 (4.28–5.58)	<0.001
Livingstone General Hospital	1.46 (0.75–2.83)	0.26	1.37 (1.20–1.57)	<0.001
Mansa General Hospital	6.86 (4.11–11.44)	<0.001	7.23 (6.36–8.24)	<0.0001
Mosi-oa-tuntya Clinic	1.51 (0.80–2.84)	0.21	2.16 (1.85–2.51)	<0.001
Ndola Central Hospital	3.91 (2.38–6.42)	<0.001	4.20 (3.70–4.77)	<0.001
Solwezi General Hospital	1.65 (0.95–2.88)	0.08	1.54 (1.34–1.76)	<0.001
St. Francis’ Hospital	1.68 (1.00–2.83)	0.05	2.05 (1.80–2.34)	<0.001
Ever smoked cigarette (^a^ 944)				
No	1.00		1.00	
Yes	1.34 (0.86–2.07)	0.19	1.37 (0.66–0.81)	<0.001
Age at sexual debut (^a^ 896)				
Less than 20 years	1.00		1.00	
20+ years	1.15 (0.89–1.49)	0.27	0.96 (0.91–1.01)	0.08
Number of sexual life partners (^a^ 639)				
5 or more	1.00		1.00	
One sexual partner	0.83 (0.62–1.12)	0.22	0.86 (0.80–0.92)	<0.001
Two sexual partners	0.98 (0.74–1.29)	0.87	1.05 (0.99–1.13)	0.10
three or four	0.97 (0.75–1.26)	0.84	1.02 (0.96–1.09)	0.53
Condom use with regular sexual partner (^a^ 2520)				
Always used a condom	1.00		1.00	
Never used a condom	1.15 (0.70–1.90)	0.57	1.08 (0.96–1.21)	0.20
Used a condom sometimes	1.38 (0.84–2.26)	0.20	1.13 (1.01–1.23)	0.03
Used a condom almost all the time	1.64 (0.89–3.02)	0.12	1.35 (1.16–1.56)	<0.001
HIV status (^a^ 3176)				
Negative	1.00		1.00	
Positive	1.91 (1.57–2.33)	<0.001	1.93 (1.84–2.02)	<0.001

^a^Number of imputed observations per variable

^b^Adjusted odds ratios (complete cases): Adjustment variables; age at screening, marital status, education, screening center, occupation, cigarette smoking, age at sexual debut, number of sexual life partners, condom use with regular sexual partner and HIV status

^c^Adjusted odds ratios (multiple imputation): Adjustment variables; age at screening, marital status, education, screening center, occupation, cigarette smoking, age at sexual debut, number of sexual life partners, condom use with regular sexual partner and HIV status

## Discussion

Findings from our study are in conformity with those from studies conducted in other parts of the world. A global perspective of the epidemiology of cancer of the cervix highlight socio-economic factors (education and income) as risk factors, and observes that education, cervical cancer screening of high risk groups and improvement in socioeconomic status can reduce cervical cancer morbidity and mortality significantly [10]. Investigations in Varanasi, India [11] also revealed that the low socio-economic status of women was significantly associated with the risk of cervical cancer (OR = 3.30, *p* < 0.001). In the United States, educational attainment was strongly and inversely associated with mortality from all cancers combined in black and white men and in white women [12]. In another investigation conducted in the United States to examine the association of breast cancer and cervical cancer incidences with income and education among whites and blacks, the incidence of cancer of the cervix showed strong negative association with education [13]. Similar findings were made in Kisumu Kenya. In this study, women who had attained some college/tertiary education had a reduced risk (AOR = 0.97; 95% CI:0.57–1.67) [14]. In another study that used data from the National Health and Nutrition Examination Survey (NHANES) for the years 2003–2010, to estimate the prevalence of genital HPV infection and explore risk factors associated with HPV infection, findings revealed that participants with only a high school degree were at a 30% increased risk of HPV infection compared to college-educated women [15]. Findings from our study and many more other studies on cervical cancer as discussed above reveal that acquiring some level of higher education is protective against abnormal cervical lesions.

A limitation of our study was that data used represented women from only 6 out of ten (10) provinces of Zambia implying that our findings cannot be generalized to Zambia as a whole but to the six provinces.

## Conclusions

We find that education was strongly and negatively associated with abnormal cervical lesions, demonstrated by presence of reduced likelihood of abnormal cervical lesions in women with primary, secondary and tertiary education. This suggests presence of differential imbalance in risk, leaning heavily to presence of higher odds of abnormality among women with no formal education and predominantly poor women mostly from rural areas. These findings may be a reflection of inherent population inequalities associated with access to and availability of primary care services such as the cervical cancer screening which seems inadequately accessible to women with no formal education who probably need it the most. On the other hand, this might also indicate serious limitations in past awareness efforts instituted both in the formative as well as during the later phases of the program. These findings therefore underline the prevailing need for urgent concerted efforts in repackaging and or repositioning cervical cancer awareness programs, targeting women with low or no formal education in whom the risk may even be higher.

## Abbreviations

AIDS: Acquired Immune Deficiency Syndrome
ANC: Antenatal Care
AOR: Adjusted Odds Ratio
CC: Cervical Cancer
CCPPZ: Cervical Cancer Prevention Program in Zambia
CI: Confidence Interval
CIDRZ: Centre for Adult Infectious Disease Research in Zambia
ERES: Research Ethics and Science (ERES) Converge
HIV: Human Immunodeficiency Virus
SPSS: Statistical Package for Social Sciences
UNZA: University of Zambia
UOR: Unadjusted Odds Ratio
VIA: Visual Inspection with Acetic-acid
